# Novel Scoring Scale for Quality Assessment of Lung Ultrasound in the Emergency Department

**DOI:** 10.5811/westjem.18225

**Published:** 2024-02-28

**Authors:** Jessica R. Balderston, Taylor Brittan, Bruce J. Kimura, Chen Wang, Jordan Tozer

**Affiliations:** * Virginia Commonwealth University Medical Center, Department of Emergency Medicine, Richmond, Virginia; † Scripps Mercy Hospital, Department of Cardiology, San Diego, California; ‡ Virginia Commonwealth University, Department of Biostatistics, Richmond, Virginia

## Abstract

**Introduction:**

The use of a reliable scoring system for quality assessment (QA) is imperative to limit inconsistencies in measuring ultrasound acquisition skills. The current grading scale used for QA endorsed by the American College of Emergency Physicians (ACEP) is non-specific, applies irrespective of the type of study performed, and has not been rigorously validated. Our goal in this study was to determine whether a succinct, organ-specific grading scale designed for lung-specific QA would be more precise with better interobserver agreement.

**Methods:**

This was a prospective validation study of an objective QA scale for lung ultrasound (LUS) in the emergency department. We identified the first 100 LUS performed in normal clinical practice in the year 2020. Four reviewers at an urban academic center who were either emergency ultrasound fellowship-trained or current fellows with at least six months of QA experience scored each study, resulting in a total of 400. The primary outcome was the level of agreement between the reviewers. Our secondary outcome was the variability of the scores given to the studies. For the agreement between reviewers, we computed the intraclass correlation coefficient (ICC) based on a two-way random-effect model with a single rater for each grading scale. We generated 10,000 bootstrapped ICCs to construct 95% confidence intervals (CI) for both grading systems. A two-sided one-sample *t*-test was used to determine whether there were differences in the bootstrapped ICCs between the two grading systems.

**Results:**

The ICC between reviewers was 0.552 (95% CI 0.40–0.68) for the ACEP grading scale and 0.703 (95% CI 0.59–0.79) for the novel grading scale (*P* < 0.001), indicating significantly more interobserver agreement using the novel scale compared to the ACEP scale. The variance of scores was similar (0.93 and 0.92 for the novel and ACEP scales, respectively).

**Conclusion:**

We found an increased interobserver agreement between reviewers when using the novel, organ-specific scale when compared with the ACEP grading scale. Increased consistency in feedback based on objective criteria directed to the specific, targeted organ provides an opportunity to enhance learner education and satisfaction with their ultrasound education.

Population Health Research Capsule What do we already know about this issue?

*A reliable method of quality assessment (QA) of ultrasound images is imperative to assess user performance and limit inconsistencies in measuring ultrasound acquisition skills.*

What was the research question?

*Is there a QA scoring scale for lung ultrasound (LUS) that is more precise than the commonly used ACEP scoring scale?*

What was the major finding of the study?

*In the QA of LUS, a novel scoring scale showed significantly more interobserver agreement compared to the ACEP scale.*

How does this improve population health?

*A more individualized scoring scale for QA of LUS results in less grading variance and more objective feedback when compared to the ACEP scale.*

## INTRODUCTION

Lung ultrasound (LUS) is frequently used in the emergency department (ED) to assess both medical and trauma patients.^1^ ^,^ ^2^ Quality assessment (QA) of ultrasound images is one of the six required elements of diagnostic ultrasound per the American College of Emergency Physicians (ACEP) and is routinely performed to evaluate image quality, ensuring appropriate patient care, and enabling reviewers to assess user performance.^2^ The use of a reliable scoring system for QA is imperative to limit inconsistencies in measuring ultrasound acquisition skills.

The current QA grading scale endorsed by ACEP was developed from a consensus report of emergency ultrasound leaders to provide a systematic method to report and communicate ultrasound findings.^2^ It is a non-specific scale that applies irrespective of the type of study performed and has not been rigorously validated. Similarly formatted organ-specific QA grading systems for cardiac and obstetric exams have been described but are not yet endorsed by ACEP and are not widely used.^2^ ^–^ ^5^ Alternative LUS assessment tools have been developed; however, they are extensive and as such impractical for routine QA use or are focused on image acquisition skills and not tailored to anatomic feedback.^6^ ^,^ ^7^ Our goal in this study was to determine whether a succinct, organ-specific grading scale designed for QA would be more precise with better interobserver agreement.

## METHODS

This was a prospective validation study of an objective QA scale for LUS. We developed a novel, lung-specific grading scale by a rigorous review of expert, published experience at an outside, unaffiliated institution (Scripps Mercy Hospital, San Diego, CA). This institution routinely performs lung imaging and has published an assessment tool for the evaluation of resident-performed bedside ultrasound B-line interpretation in thoracic ultrasound, as well as an analogous cardiac quality assessment scale.^3^ ^,^ ^7^ ^–^ ^13^ In the expert review, the current available, organ-specific grading scale found in the literature was modified to the anatomy of the chest wall.^3^ ^,^ ^5^ The gradations of the scale were empirically derived from the experience at this institution in addition to a rigorous review of the literature. ^3^ ^,^ ^5^ ^,^ ^7^ ^–^ ^13^ The use of four critical landmarks—rib shadows, pleural line, A/B lines, and technical flaws—were recognized as commonalities in all published images in LUS studies, including expert consensus.^14^ ^,^ ^15^ We, therefore, divided these landmarks into a point scale that progressively defines the pattern of acquisition required to obtain an image (ie, bones first, pleural line, followed by artifacts). We described technical flaws as non-optimized depth/gain, distracting adjacent structures, inadequate axis, or hand movement. We deemed flaws to be major if they were present to a degree significant enough to decrease diagnostic capabilities, or if multiple flaws were present.

The scale was then validated at an urban academic tertiary care center in Richmond, Virginia. We identified the first 100 LUS studies completed as part of regular clinical practice in the ED by emergency physicians with two or more LUS videos performed in the year 2020. Dedicated thoracic ultrasound examinations are in general performed by resident physicians with attending oversight. Studies were obtained using Sonosite X Porte ultrasound machine (Fujifilm Sonosite, Bethell, WA) using either the C60XP 5-2-MHz curvilinear transducer, L25 13-6-MHz linear array transducer or the P19 5-1-MHz phased array probe. Four reviewers who were either emergency ultrasound fellowship-trained or current fellows with at least six months of QA experience scored each of the 100 studies resulting in a total of 400. Two blinded reviewers used the current ACEP grading scale,^2^ and two used a novel lung-specific grading scale; there was one fellow and one ultrasound-trained physician in each group (Figure). The primary outcome was the level of agreement between the reviewers, indicating the reliability of the scoring system. Our secondary outcome was the variability of the scores given to the studies. For the agreement between reviewers, we computed the intraclass correlation coefficient (ICC) based on two-way random-effect model with a single rater for each grading scale. Ten thousand bootstrapped ICCs were generated to construct 95% confidence intervals (CI) for both grading systems. We used a two-sided one-sample *t*-test to determine whether there were differences in the bootstrapped ICCs between the two grading systems.

**Figure.**
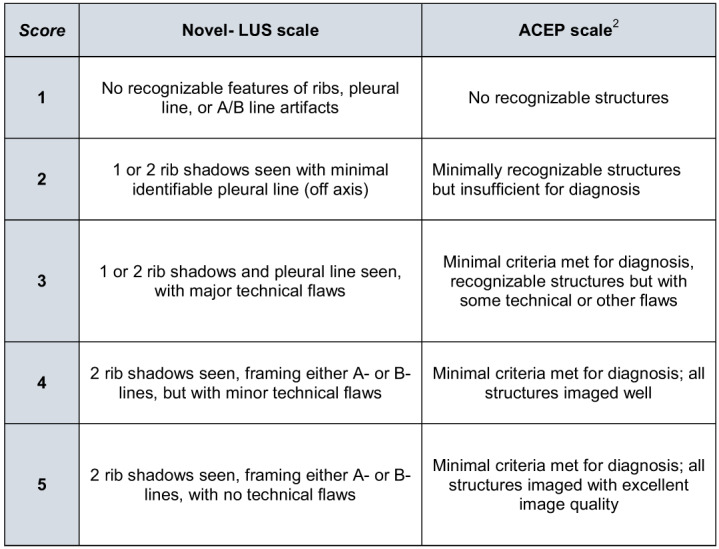
Comparison of the novel, lung ultrasound quality assessment scale with the traditional American College of Emergency Physicians scale. *ACEP*, American College of Emergency Physicians; *LUS*, lung ultrasound.

## RESULTS

The first 100 LUS studies completed in the ED by emergency medicine residents (postgraduate year [PGY]-1, 42%; PGY-2, 14%; PGY-3, 22%) and ED faculty (22%) were reviewed by four blinded reviewers. Images were obtained using the linear probe (27%), curvilinear probe (32%), phased array probe (28%), or a combination of probes (13%). Studies had a median of six clips (IQR 4–9). The scores given using the ACEP scale and the novel scale are summarized in the Table. The ICC between reviewers was 0.552 (95% CI 0.4–0.68) for the ACEP grading scale and 0.703 (95% CI-0.59, 0.79) for the novel grading scale (*P* < 0.001), indicating significantly more interobserver agreement using the novel scale compared to the ACEP scale. The variance of scores was similar (0.93 and 0.92 for the novel and ACEP scales, respectively).

**Table.** Summary table of scoring systems.

Statistics	Novel	ACEP
N^*^	200	200
Min, max	1, 5	1, 5
Mean (SD)	3.70 (0.96)	3.32 (0.96)
Median	4	3
Q1, Q3	3, 4	3, 4
ICC (95% CI)	0.703 (0.59, 0.79)	0.552 (0.40, 0.68)
Variance	0.93	0.92
Variance ratio (Novel: ACEP)	1.01	

* N = number of scores given.

*ACEP*, American College of Emergency Physicians; *ICC*, intraclass correlation coefficient; *CI*, confidence interval.

## DISCUSSION

The current ACEP grading scale used for QA was developed from a consensus report of emergency ultrasound leaders but has not been systematically validated.^2^ The use of a reliable, validated scoring system for QA is imperative to limit inconsistencies and ensure objectivity in measuring ultrasound acquisition skill. The vague language used in the ACEP scale may contribute to variable interpretation by those assessing studies, leading to discrepancies in grading ultrasound skill. Inconsistent feedback may confuse the learner and hinder growth of technical skill. In our study, we found that there was an increased interobserver agreement between reviewers when using the novel, organ-specific scale when compared with the ACEP grading scale. Increased consistency in feedback, combined with directed feedback to the specific targeted organ, provides an opportunity to enhance learner education and satisfaction with their ultrasound education.

Organ-specific cardiac and obstetric QA grading systems have been described, although they have not yet been widely adopted in clinical practice.^3^ ^–^ ^5^ This is thought to be due in part to the complexity of these scales and/or that they were validated outside the ED, limiting the external validity.^3^ ^,^ ^4^ ^,^ ^6^ ^,^ ^7^ We sought to develop a scale that was concise, organ-specific, and applicable to the most common setting in which LUS is performed. To improve such vague language as “all structures imaged well,” we found benefit in specifically stating the anatomic landmarks needed to maximize diagnostic imaging in each view. By emphasizing proper imaging technique before diagnostic interpretation, our assessment tool may improve errors in image grading and reduce learner feedback variability.

## LIMITATIONS

Our study was limited by its evaluation of a QA experience at a single, academic tertiary-care center in which the validation took place. Patient demographics were not collected. The blinded reviewers all trained (or current trainees) at the same clinical ultrasound fellowship and, therefore, were taught to perform QA using the ACEP grading scale in a similar manner. Interestingly, this perhaps may have contributed to a higher agreement with the ACEP scale than if, alternatively, reviewers had trained at different institutions. Further, the scale itself was developed after an extensive review of the literature, customized into a feasible scale that is directly applicable to learner objectives. As such, this scale lacks the rigor of alternative methodological methods such as modified Delphi analysis. Importantly, this scale did not validate whether the score was related to the diagnosis or outcome, or whether it improved QA efficiency or educational feedback, but rather the degree of agreement. Additionally, our scale focuses on pathology related to the pleural line itself and does not include language to assess the ability to diagnose a pleural effusion. Finally, our study involved reviewers with six months experience in QA and included a small (100) number of studies; consequently, our results may be understated. Further research is warranted to validate this novel scale, investigate learner satisfaction, and assess its impact on educational enhancement.

## CONCLUSION

We found that a more individualized quality assessment scale of ultrasound imaging targeted to a specific organ—in this case the lung—results in less grading variance and more consistent, objective feedback. This finding may have implications on knowledge gained and learner satisfaction. Future studies are warranted prior to the adoption of this novel scale in clinical practice.

## References

[1] American College of Surgeons’ Committee on Trauma *Advanced Trauma Life Support* (ATLS) ; 10^th^ ed. Chicago, Illinois: American College of Surgeons; 2018.

[2] Ultrasound guidelines: emergency , point-of-care and clinical ultrasound guidelines in medicine. Ann Emerg Med. 2017;69(5):e27–54. 28442101 10.1016/j.annemergmed.2016.08.457

[3] Kimura BJ Gilcrease GW Showalter BK et al . Diagnostic performance of a pocket-sized ultrasound device for quick-look cardiac imaging. Am J Emerg Med. 2012;30(1):32–6. 21035983 10.1016/j.ajem.2010.07.024

[4] Salomon LJ Nassar M Bernard JP et al . A score-based method to improve the quality of emergency gynaecological ultrasound examinations. Eur J Obstet Gynecol Reprod Biol. 2009;143(2):116–20. 19203825 10.1016/j.ejogrb.2008.12.003

[5] DeMasi S Taylor LA Weltler A et al . Novel quality assessment methodology in focused cardiac ultrasound. Acad Emerg Med. 2022;29(10):1261–3. 35842913 10.1111/acem.14562 PMC9804740

[6] Ravishankar SM Tsumura R Hardin JW et al . Anatomical feature-based lung ultrasound image quality assessment using deep convolutional neural network. IEEE Int Ultrason Symp. 2021;2021: 10.1109/ius52206.2021.9593662. PMC9373065 35966447

[7] Dessie AS Calhoun AW Kanjanauptom P et al . Development and validation of a point-of-care ultrasound image quality assessment tool: the POCUS IQ scale. Ann Emerg Med. 2019;74(4):S45–6. 36165271 10.1002/jum.16095

[8] Garibyan VN Amundson SA Shaw DJ et al . The prognostic value of lung ultrasound findings in hospitalized patients undergoing echocardiography. J Ultrasound Med. 2018;37(7):1641–8. 29266328 10.1002/jum.14511

[9] Kimura BJ Lou MM Dahms EB et al . Prognostic implications of a point-of-care ultrasound examination upon hospital admission. J Ultrasound Med. 2020;39(2):289–97. 31378976 10.1002/jum.15102

[10] Kimura BJ Nayak KR . “Asymptomatic” flash pulmonary edema by point-of-care ultrasound: a novel bedside finding of transient global ischemia. JACC Case Rep. 2020;2(10):1545–9. 34317014 10.1016/j.jaccas.2020.06.029 PMC8302159

[11] Kimura BJ Shi R Tran EM et al . Outcomes of simplified lung ultrasound exam in COVID-19: Implications for self-imaging. J Ultrasound Med. 2022;41(6):1377–84. 34473363 10.1002/jum.15820 PMC8661724

[12] Kimura BJ Resnikoff PM Tran EM et al . Simplified lung ultrasound examination and telehealth feasibility in early SARS-CoV-2 Infection. J Am Soc Echocardiogr. 2022;35(10):1047–54. 35691456 10.1016/j.echo.2022.05.015 PMC9183238

[13] Mai TV Shaw DJ Amundson SA et al . Learning to apply the pocket ultrasound device on the critically ill: comparing six “quick look” signs for quality and prognostic values during initial use by novices. Critical Care. 2013;17(5):448. 10.1186/cc12875 PMC4056255 24004510

[14] Taylor A Anjum F O’Rourke MC . Thoracic and lung ultrasound. In: *StatPearls* [Internet]. Treasure Island, FL: StatPearls Publishing; 2022. 29763189

[15] Mayo PH Copetti R Feller-Kopman D et al . Thoracic ultrasonography: a narrative review. Intensive Care Med. 2019;45(9):1200–11. 31418060 10.1007/s00134-019-05725-8

